# A Review of Adult Mortality Due to 2009 Pandemic (H1N1) Influenza A in California

**DOI:** 10.1371/journal.pone.0018221

**Published:** 2011-04-05

**Authors:** Janice K. Louie, Cynthia Jean, Meileen Acosta, Michael C. Samuel, Bela T. Matyas, Robert Schechter

**Affiliations:** California Department of Public Health, Richmond, California, United States of America; University of Hong Kong, Hong Kong

## Abstract

**Background:**

While children and young adults had the highest attack rates due to 2009 pandemic (H1N1) influenza A (2009 H1N1), studies of hospitalized cases noted high fatality in older adults. We analyzed California public health surveillance data to better characterize the populations at risk for dying due to 2009 H1N1.

**Methods and Findings:**

A case was an adult ≥20 years who died with influenza-like symptoms and laboratory results indicative of 2009 H1N1. Demographic and clinical data were abstracted from medical records using a standardized case report form. From April 3, 2009 – August 10, 2010, 541 fatal cases ≥20 years with 2009 H1N1 were reported. Influenza fatality rates per 100,000 population were highest in persons 50–59 years (3.5; annualized rate = 2.6) and 60–69 years (2.3; annualized rate = 1.7) compared to younger and older age groups (0.4–1.9; annualized rates = 0.3–1.4). Of 486 cases hospitalized prior to death, 441 (91%) required intensive care unit (ICU) admission. ICU admission rates per 100,000 population were highest in adults 50–59 years (8.6). ICU case-fatality ratios among adults ranged from 24–42%, with the highest ratios in persons 70–79 years. A total of 425 (80%) cases had co-morbid conditions associated with severe seasonal influenza. The prevalence of most co-morbid conditions increased with increasing age, but obesity, pregnancy and obstructive sleep apnea decreased with age. Rapid testing was positive in 97 (35%) of 276 tested. Of 482 cases with available data, 384 (80%) received antiviral treatment, including 49 (15%) of 328 within 48 hours of symptom onset.

**Conclusions:**

Adults aged 50–59 years had the highest fatality due to 2009 H1N1; older adults may have been spared due to pre-existing immunity. However, once infected and hospitalized in intensive care, case-fatality ratios were high for all adults, especially in those over 60 years. Vaccination of adults older than 50 years should be encouraged.

## Introduction

The 2009 pandemic (H1N1) influenza A virus (2009 H1N1) emerged in April 2009 to cause a global pandemic that lasted 16 months [1]. The highest attack rates were reported among children and young adults; 60% of laboratory-confirmed infections and 32–45% of hospitalized cases in the United States occurred in persons under 18 years of age, and cases younger than 65 years accounted for approximately 90% of deaths [1]. These preliminary data led to initial ACIP recommendations in July 2009 to target limited initial supplies of H1N1 vaccine to persons younger than age 65 years [2]; these recommendations have since been updated [3].

In April 2009 the California Department of Public Health (CDPH) initiated surveillance for hospitalized and fatal cases of 2009 H1N1 infection. Our early review of the first 16 weeks of the pandemic suggested that while most hospitalized cases were young, the case-fatality ratio among hospitalized cases was highest in those aged 50 years or older [4]. In this report, we expand on our previous findings to review the epidemiologic and clinical characteristics of adult Californians who died due to 2009 H1N1 throughout the pandemic.

## Materials and Methods

### Ethics Statement

During the period of this study 2009 H1N1 infection was a reportable disease in California. This activity was reviewed and determined by the California Committee for the Protection of Human Subjects to be consistent with an emergent public health response that did not require institutional review board approval.

A case was an adult ≥20 years of age who died with clinical illness consistent with respiratory infection and had results from testing of a respiratory specimen that were indicative of 2009 H1N1 influenza by a reverse-transcriptase polymerase chain reaction assay developed by the Centers for Disease Control and Prevention and authorized by the FDA (http://www.cdc.gov/h1n1flu/guidance/rapid_testing.htm). During April 3 - August 11, 2009, state recommendations for laboratory testing as well as case-based reporting was performed for all Californians who were hospitalized or died with 2009 H1N1 infection. After August 11, 2009, state recommendations for laboratory testing and case-based reporting were limited to Californians with 2009 H1N1 infection who required intensive care unit (ICU) admission or died. Hospitalized and fatal cases were reported by providers and hospitals to local health departments. A few cases that died outside of hospitals were reported by county coroners to the local health department. Cause of death was determined by review of death certificates by the reporting clinician or local health department. All cases were subsequently reported from local health departments to CDPH. For both hospitalized and fatal cases, data on demographics, including race/ethnicity, clinical presentation and course, co-morbid conditions, and laboratory and radiographic findings were abstracted from medical records, and autopsy reports where appropriate, by staff at the local health department or CDPH using the same standardized case report form. Body mass index (BMI) was calculated from available height and weight data as body weight in kilograms divided by the square of height in meters. Adult obesity was defined in accordance with the National Institutes of Health (NIH) classification [5]. Among reported cases with BMI data, we analyzed: NIH BMI categories of 30–39 (obese) and ≥40 (extreme obesity) [5]. Co-morbidities were considered absent in cases where records stated that the patient was previously healthy or had no underlying medical conditions; likewise, only the 454 cases with available height and weight data were included in the analysis of BMI and association with age.

Population-based fatality rates were calculated by age group using all reported influenza deaths, and were defined as number of fatal cases per 100,000 population. Case-fatality ratios were calculated using reported deaths that had been admitted to the ICU and were defined as (number of fatal ICU cases from age group/total number of ICU cases within that age group)*100. Distributions of demographic and clinical risk factors among age groups were also analyzed. Significant trends among different age groups were calculated using the Cochran-Armitage trend test. For the trend analysis, the denominators used for extreme obesity (BMI ≥40) and obesity (BMI 30–39) were 454 (all cases with height and weight data available) and 356, respectively; in the latter, extremely obese cases (n = 98) were excluded in order to assess any association between age and the presence of obesity (BMI 30–39) as compared to the absence of obesity (BMI<30). All analyses were performed using SAS 9.2 (SAS Institute, Cary, NC).

## Results

### Case fatality rates

Between April 3, 2009 – August 10, 2010, 541 fatal cases of 2009 H1N1 in adults ≥20 years of age were reported. Table 1 presents data on ICU admission and mortality by age group, and includes age groups under 20 years for comparison. Influenza fatality rates were highest in persons 50–59 years (3.5; annualized rate 2.6) and 60–69 years (2.3; annualized rate 1.7) and lower (0.4–1.9; annualized rates 0.3–1.4) in younger and older age groups. ICU admission rates per 100,000 population were highest in adults 50–59 years of age (8.6). Case-fatality ratios in adults ranged from 24–42%, with the highest ratio in persons 70–79 years.

**Table 1:** Total number of cases reported and fatality rate of 2009 H1N1 influenza in California, April 3, 2009 - August 10, 2010.

	FATAL CASES			ICU CASES			
Age, y	Number of fatal cases	Influenza fatality rate per 100,000 population^a^	Annual influenza fatality rate per 100,000 population^a^	Number of cases admitted to ICU	ICU admission rate per 100,000 population^a^	Number of fatal cases admitted to ICU	Case-fatality ratio^b^
<1	7	1.2	0.9	83	14.8	7	8.4%
1–9	21	0.4	0.3	284	5.8	18	6.3%
10–19	27	0.5	0.3	216	3.7	25	11.6%
20–29	77	1.4	1.1	268	4.9	64	23.9%
30–39	72	1.4	1.0	208	4.0	53	25.5%
40–49	108	1.9	1.4	283	4.9	89	31.4%
50–59	170	3.5	2.6	425	8.6	142	33.4%
60–69	72	2.3	1.7	164	5.2	62	37.8%
70–79	27	1.5	1.1	52	2.9	22	42.3%
80+	15	1.2	0.9	25	2.1	9	36.0%
Total	596	1.5	1.2	2008	5.2	491	24.5%

Abbreviations: ICU, intensive care unit.

a Rates were calculated using 2009 statewide age-specific population projections published by the State of California Department of Finance (URL: http://www.dof.ca.gov/research/demographic/data/race-ethnic/2000-50/).

b Excludes non-hospitalized fatalities; the case-fatality ratio is calculated using the following formula: (number of fatal ICU cases from age group/total number of ICU cases within that age group)*100.

**Figure 1:**
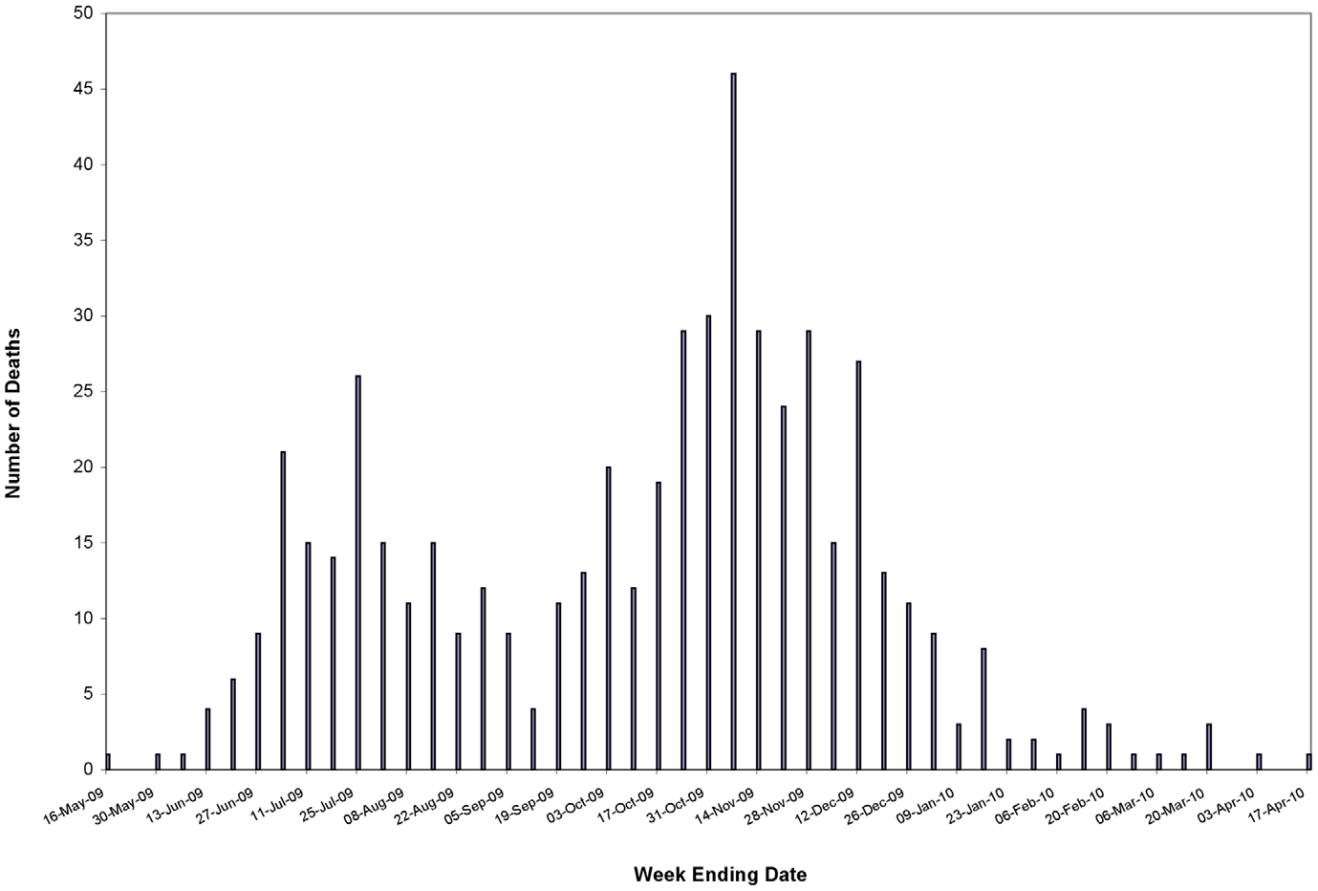
Fatal cases of 2009 pandemic (H1N1) influenza A in California by date of death. In California, mortality peaks due to 2009 pandemic (H1N1) influenza A coincided with two waves of 2009 H1N1 activity: April 3-October 3, 2009, and October 4, 2009-February 4, 2010. The influenza fatality rate remained highest for 50–59 year-olds during both time periods.

To identify if there was any temporal variation in mortality, influenza fatality rates and case-fatality ratios were calculated for two separate time periods that corresponded to two waves of 2009 H1N1 activity: April 3-October 3, 2009, and October 4, 2009-February 4, 2010 (Figure 1). The influenza fatality rate remained highest for 50–59 year-olds during both time periods: 1.1 (range: 0.2–1.1; 0.6 for all ages) during the first wave, and 1.8 (range: 0.2–1.8; 0.8 for all ages) during the second wave. Likewise, case-fatality ratios were similarly high among adults admitted to the ICU during both time periods.

### Epidemiologic and clinical characteristics of fatal cases

Table S1 summarizes epidemiologic and clinical characteristics. Of the 541 fatal cases ≥20 years of age, 267 (49%) were male. Of 519 fatal cases with available information, 224 (43%) were Hispanic and 210 (40%) were non-Hispanic white.

The median time from onset of symptoms to death was 14 days (range 0–131 days). Of the 541 fatalities, 486 (90%) were hospitalized. 441 (91%) hospitalized cases required intensive care. 399 (95%) of 422 received mechanical ventilation.

In 276 fatal cases where rapid testing was performed, 97 (35%) tested positive. Sixty-nine (13%) had also microbiologic evidence of secondary bacterial infection.

Of 482 cases where data were available, 384 (80%) received antiviral treatment; the median time in days from onset to antiviral treatment was 5 days (range: 0–47 days). Forty-nine (15%) of 328 received antiviral treatment within 48 hours of symptom onset.

Fifty-five adults died at home without hospitalization; of these, 32 (58%) had been seen as an outpatient prior to their death. Of the 40 of 55 cases who had information available, nine (23%) had received antiviral treatment prior to death. Dates of treatment were available for eight of these cases; the median time from onset of symptoms to treatment was 3 days (range 1–10 days), and three (38%) had received treatment within 48 hours of symptom onset.

### Co-morbid conditions associated by the Advisory Committee on Immunization Practices (ACIP) with severe influenza

Four hundred twenty-five (80%) had a co-morbid condition associated by the ACIP with severe seasonal influenza infection (3); the most common were chronic metabolic disease, primarily diabetes mellitus and renal disease (215/531; 40%), chronic lung disease (202/530; 38%), chronic cardiac disease (143/521; 27%), immunosuppressive conditions, primarily neoplasms (133/529; 25%) and extreme obesity with a BMI ≥40 (98/454; 22%). Of 532 with available data, 364 (68%) fatal cases had other comorbidity such as obesity [body mass index 30–39 (159/454;35%)] and hypertension (208/529; 39%). There was no association between the number of co-morbidities in each case and median time from onset of symptoms to death.

Table S1 also demonstrates the distribution of co-morbid illnesses and details of hospital course by age group. Older fatal cases were significantly more likely than younger cases to have ACIP-defined high risk conditions (p<0.001), including chronic lung diseases (especially chronic obstructive pulmonary disease; p<0.0001), chronic cardiac diseases (coronary artery disease, congestive heart failure and arrythmias; p<0.0001), immunosuppression (mainly malignancy, solid organ transplant, or chronic immunosuppressive drugs; p<0.0001), and chronic neurologic diseases (history of cerebrovascular accident, Alzheimer's disease, Parkinson's disease and dementia; p<0.05) (7). In patients younger than 70 years, the prevalence of chronic metabolic diseases (diabetes mellitus, p<0.0001; renal disease, p<0.05, and hypothyroidism, p<0.05) and hyperlipidemia (p<0.0001) significantly increased with age. Older fatal cases were also significantly more likely than younger cases to have conditions not associated by the ACIP with severe influenza, including chronic gastrointestinal disease (p<0.0001) and hypertension (p<0.0001).

Conversely, the prevalence of certain ACIP-defined high risk conditions significantly decreased with increasing age, including extreme obesity (BMI ≥40) (p<0.0001), pregnancy (p<0.0001) and obstructive sleep apnea (p<0.05). The prevalence of obesity (BMI 30–39), which was highest in adults aged 40–49 years, significantly decreased with increasing age in persons older than that age group (p = 0.0007).

## Discussion

We reviewed more than 500 fatal cases of 2009 H1N1 in California to better characterize populations at risk. Persons aged 50–59 years had the highest influenza fatality rate. Over 90% of fatal cases had co-morbid illnesses; 80% had a co-morbid condition associated by the ACIP with severe seasonal influenza infection [3]. Age groups older than 60 years of age had progressively decreasing influenza fatality rates despite their increasing prevalence of co-morbidity associated with severe influenza; the most common co-morbidity in adults under age 60 was obesity. Although all adult age groups had high case-fatality ratios once admitted to intensive care, persons 70–79 years were most likely (42%) to die. While cases were ascertained from passive reporting by clinicians, who may have underreported cases due to under-recognition or limited access to confirmatory laboratory testing, these limitations are unlikely to have introduced any selection bias that would affect these findings.

Others have reported maximal case fatality rates in different age groups. During the first wave of the pandemic the case fatality rate in Mexico ranged from 3 to 13 per 100,000, with the highest rates occurring in persons aged 20–49 years of age [6]. In the same period adults aged 40–59 years in North, Central and South America, Europe and Asia demonstrated the highest mortality at 0.54–0.57 per million population, compared to 0.40 per million for all ages [7]. In England, public health surveillance data collected over two pandemic waves found higher population mortality rates in persons ≥65 years (9.3 per 1000) compared to under 65 years (0.4 per 1000) [8]. Demography may have contributed to these differences, as it is possible that higher attack rates occurred in larger age segments of the population. The variability in mortality by setting may also reflect differences in ascertainment criteria, or testing and surveillance methods. Additionally, susceptibility to severe infection may have varied by age at different times during the pandemic as immunity spread within a population. Our results are consistent with US national data (which includes California), where cases aged 50–64 years had the highest death rate (1.69 per 100,000, compared to 0.97 for all age groups) (http://www.cdc.gov/H1N1flu/hosp_deaths_ahdra.htm). Even with this variation, all studies have estimated mortality rates due to 2009 H1N1 that are no higher than those typically reported for seasonal influenza [3]; these rates are likely underestimates due to underreporting.

With seasonal influenza, persons ≥65 years account for >95% of deaths; those over 85 years are 32 times more likely to die than persons aged 60–65 years [3]. The decline in 2009 H1N1 case-fatality rates we observed in persons ≥60 years, despite an increasing prevalence with age of ACIP-defined high risk conditions, strongly suggests that pre-existing immunity provided some protection. This immunity was likely derived from exposure to antigenically-related H1N1 viruses that circulated between emergence of the pandemic H1N1 influenza virus in 1918 and the H2N2 virus in 1957 [9]. Serologic studies support this hypothesis: one-third of those born in the US before 1950 had high titers against 2009 H1N1 prior to 2009, compared to 4% of persons born after 1980 [9]. Cross-protective antibody was found in 96% of Finns born between 1909–1919, 14–77% born between1920–1944, and few born after 1944 [10].

Regardless of pre-existing immunity, fatality of all adults hospitalized in intensive care for 2009 H1N1 was high, including in persons over 60 years. The vulnerability of the elderly to severe illness from a newly emergent influenza virus should not be underestimated. Experience with previous pandemics suggests that the younger age bias observed for 2009 H1N1 may shift in coming months to older persons. While half of influenza-related deaths during the 1968-69 influenza A (H3N2) pandemic and a large proportion of deaths during the 1957-58 influenza A (H2N2) and the 1918–1919 influenza A (H1N1) pandemics occurred among persons <65 years of age, this group accounted for progressively smaller proportions of deaths during the decade following each pandemic [11]–[12]. These observations reinforce the importance of routine vaccination of older age groups, who appear to respond well to monovalent vaccination against 2009 H1N1 [13]–[14]. While the overall efficacy of vaccination in the elderly is uncertain, one large randomized controlled trial found that vaccination for seasonal influenza decreased the likelihood of hospitalization by 27% and death by 50% in the elderly residing in the community [15].

We conclude with two major points: in contrast to seasonal influenza, adults aged 50–59 years in California had the highest fatality rate due to 2009 H1N1; adults over 60 years may have been somewhat spared due to pre-existing relative immunity. However, once infected and hospitalized in intensive care, case-fatality ratios in California were high for all adults, especially the elderly. When 2009 H1N1 is known to be circulating, clinicians should maintain a high level of suspicion in persons of all ages presenting with influenza-like illness, including middle aged and elderly adults. Additionally, when novel pandemic influenza viruses emerge and cause disease, immunization policies, particularly during shortages, should be flexible and consider the changing demographic of those affected, including taking into account those populations with high mortality rates.

## Supporting Information

Table S1 Co-morbid illnesses by age group in fatal cases of 2009 pandemic (H1N1) influenza A reported in California, April 2009-August 2010.
(DOC)
