# Supplementary material for: A Review of Adult Mortality Due to 2009 Pandemic (H1N1) Influenza A in California
Source: PLoS One. 2011 Apr 5;6(4):e18221. doi: 10.1371/journal.pone.0018221 (PMC3071719; doi:10.1371/journal.pone.0018221)
Supplement: Table S1 — Co-morbid illnesses by age group in fatal cases of 2009 pandemic (H1N1) influenza A reported in California, April 2009-August 2010. (DOC) [file pone.0018221.s001.doc]

**Table S1. Selected underlying medical conditions by age group, fatal cases of 2009 H1N1 influenza in adults 20 years and older, California, April 3, 2009 - August 10, 2010**

|  |  |  | **Total** | **20 - 39 y** | **40 – 49 y** | **50 – 59 y** | **60 - 69 y** | **≥ 70 y** | **Trend Test** |
| --- | --- | --- | --- | --- | --- | --- | --- | --- | --- |
|  |  |  | **No. (%)** | **No. (%)** | **No. (%)** | **No. (%)** | **No. (%)** | **No. (%)** | ***P* value** |
| Number |  |  | 541 | 149 | 108 | 170 | 72 | 42 |  |
| Sex | Male |  | 267 (49) | 71 (48) | 58 (54) | 87 (51) | 37 (51) | 14 (33) | NS |
| Race/Ethnicity | | |  |  |  |  |  |  | p=0.0034* |
|  | Non-Hispanic White | | 210/519 (40) | 36/144 (25) | 42/105 (40) | 81/159 (51) | 34/70 (49) | 17/41 (41) |  |
|  | Hispanic | | 224/519 (43) | 80/144 (56) | 48/105 (46) | 58/159 (36) | 21/70 (30) | 17/41 (41) |  |
|  | Asian/ Pacific Islander | | 40/519 (8) | 14/144 (10) | 6/105 (6) | 7/159 (4) | 8/70 (11) | 5/41 (12) |  |
|  | Black |  | 35/519 (7) | 11/144 (8) | 5/105 (5) | 11/159 (7) | 7/70 (10) | 1/41 (2) |  |
|  | Native American | | 5/519 (1) | 2/144 (1) | 3/105 (3) | 0/159 (0) | 0/70 (0) | 0/41 (0) |  |
|  | Other |  | 5/519 (1) | 1/144 (1) | 1/105 (1) | 2/159 (1) | 0/70 (0) | 1/41 (2) |  |
| Co-morbid conditions associated with severe influenza^†^ | | | 425/533 (80) | 113/148 (76) | 74/105 (70) | 133/166 (80) | 64 (89) | 41 (98) | p=0.0003 |
|  | Chronic lung disease | | 202/530 (38) | 40/145 (28) | 33/105 (31) | 66/166 (40) | 33 (46) | 30 (71) | p<0.0001 |
|  |  | Asthma | 85/530 (16) | 22/145 (15) | 22/105 (21) | 24/166 (14) | 9 (13) | 8 (19) | NS |
|  |  | COPD | 89/530 (17) | 1/145 (1) | 10/105 (10) | 36/166 (22) | 18 (25) | 24 (57) | p<0.0001 |
|  |  | Obstructive sleep apnea | 32/530 (6) | 13/145 (9) | 7/105 (7) | 9/166 (5) | 2 (3) | 1 (2) | p=0.0324 |
|  |  | Other/unknown^‡^ | 57/530 (11) | 10/145 (7) | 9/105 (9) | 15/166 (9) | 12 (17) | 11 (26) | p=0.0005 |
|  | Chronic cardiac disease | | 143/521 (27) | 20/144 (14) | 21/104 (20) | 51/163 (31) | 29/70 (41) | 22/40 (55) | p<0.0001 |
|  |  | Congestive heart failure | 85/521 (16) | 10/144 (7) | 9/104 (9) | 36/163 (22) | 19/70 (27) | 11/40 (28) | p<0.0001 |
|  |  | Coronary arterial disease | 53/521 (10) | 3/144 (2) | 7/104 (7) | 19/163 (12) | 15/70 (21) | 9/40 (23) | p<0.0001 |
|  |  | Arrythmia | 40/521 (8) | 3/144 (2) | 6/104 (6) | 14/163 (9) | 7/70 (10) | 10/40 (25) | p<0.0001 |
|  | Metabolic disease | | 215/531 (40) | 34/147 (23) | 44/104 (42) | 74/166 (45) | 42 (58) | 21 (50) | p<0.0001 |
|  |  | Diabetes mellitus | 131/531 (25) | 13/147 (9) | 28/104 (27) | 52/166 (31) | 26 (36) | 12 (29) | p<0.0001 |
|  |  | Renal disease | 88/531 (17) | 14/147 (10) | 15/104 (14) | 28/166 (17) | 25 (35) | 6 (14) | p=0.0014 |
|  |  | Hypothyroidism | 52/531 (10) | 8/147 (5) | 8/104 (8) | 17/166 (10) | 13 (18) | 6 (14) | p=0.0037 |
|  |  | Other/unknown^§^ | 19/531 (4) | 5/147 (3) | 4/104 (4) | 4/166 (2) | 5 (7) | 1 (2) | NS |
|  | Immunosuppressed | | 133/529 (25) | 25/146 (17) | 22/104 (21) | 37/166 (22) | 30/71 (42) | 19 (45) | p<0.0001 |
|  |  | Cancer/transplant/drugs^¶^ | 115/529 (22) | 21/146 (14) | 16/104 (15) | 31/166 (19) | 29/71 (41) | 18 (43) | p<0.0001 |
|  |  | HIV/AIDS | 13/529 (2) | 3/146 (2) | 7/104 (7) | 3/166 (2) | 0/71 (0) | 0 (0) | NS |
|  |  | Other/unknown^#^ | 25/529 (5) | 7/146 (5) | 3/104 (3) | 10/166 (6) | 3/71 (4) | 2 (5) | NS |
|  | Neuromuscular disorder | | 88/526 (17) | 21/145 (14) | 14/104 (13) | 23/164 (14) | 18/71 (25) | 12 (29) | p=0.0147 |
|  |  | Seizure disorder | 27/526 (5) | 9/145 (6) | 6/104 (6) | 5/164 (3) | 6/71 (8) | 1 (2) | NS |
|  |  | CP/ developmental delay | 27/526 (5) | 11/145 (8) | 6/104 (6) | 5/164 (3) | 5/71 (7) | 0 (0) | NS |
|  |  | Cerebrovascular accident | 16/526 (3) | 1/145 (1) | 1/104 (1) | 6/164 (4) | 5/71 (7) | 3 (7) | p=0.0018 |
|  |  | Alzheimer's/PD/dementia | 10/526 (2) | 0/145 (0) | 1/104 (1) | 1/164 (1) | 3/71 (4) | 5 (12) | p<0.0001 |
|  |  | Other/unknown^**^ | 31/526 (6) | 7/145 (5) | 6/104 (6) | 9/164 (5) | 5/71 (7) | 4 (10) | NS |
|  | Hemoglobinopathy | | 2/528 (<1) | 0/145 (0) | 1/104 (1) | 1/165 (1) | 0 (0) | 0 (0) | NS |
|  | Pregnancy | | 16/254 (6) | 15/74 (20) | 1/46 (2) | 0/74 (0) | 0/34 (0) | 0/26 (0) | p<0.0001 |
|  | Extreme obesity (BMI ≥40)^††^ | | 98/454 (22) | 46/122 (38) | 18/88 (20) | 27/145 (19) | 6/64 (9) | 1/35 (3) | p<0.0001 |
| Other co-morbid illness^†^ | | | 364/532 (68) | 67/146 (46) | 76/105 (72) | 125/167 (75) | 62 (86) | 34 (81) | p<0.0001 |
|  | Obesity (BMI 30-39)^††^ | | 159/356 (45) | 35/76 (46) | 43/70 (61) | 58/118 (49) | 15/58 (26) | 8/34 (24) | p=0.0007 |
|  | Gastrointestinal disease | | 119/521 (23) | 13/143 (9) | 24/104 (23) | 41/161 (25) | 24/71 (34) | 17 (40) | p<0.0001 |
|  |  | GERD | 36/521 (7) | 3/143 (2) | 7/104 (7) | 10/161 (6) | 7/71 (10) | 9 (21) | p<0.0001 |
|  |  | Other/unknown^‡‡^ | 95/521 (18) | 11/143 (8) | 19/104 (18) | 33/161 (21) | 22/71 (31) | 10 (24) | p<0.0001 |
|  | Hyperlipidemia | | 88/531 (17) | 5/146 (3) | 13/105 (12) | 37/167 (22) | 23/71 (32) | 10 (24) | p<0.0001 |
|  | Hypertension | | 208/529 (39) | 26/144 (18) | 33/105 (31) | 78/166 (47) | 42 (58) | 29 (69) | p<0.0001 |
| Clinical Findings and Course | | |  |  |  |  |  |  |  |
|  | Positive rapid test result | | 97/276 (35) | 20/73 (27) | 16/46 (35) | 35/94 (37) | 17/37 (46) | 9/26 (35) | NS |
|  | Infiltrates on CXR or CT | | 463/483 (96) | 114/122 (93) | 94/97 (97) | 150/153 (98) | 67/71 (94) | 38/40 (95) | NS |
|  | Hospitalized ≥24 h | | 486 (90) | 123 (83) | 95 (88) | 158 (93) | 70 (97) | 40 (95) | p=0.0001 |
|  |  | Admitted to ICU | 441/486 (91) | 117/123 (95) | 89/95 (94) | 142/158 (90) | 62/70 (89) | 31/40 (78) | p=0.0011 |
|  |  | Mechanical ventilation | 399/422 (95) | 112/114 (98) | 79/84 (94) | 136/139 (98) | 55/58 (95) | 17/27 (63) | p<0.0001 |
|  | Antiviral treatment | | 384/482 (80) | 105/135 (78) | 80/98 (82) | 120/151 (79) | 50/63 (79) | 29/35 (83) | NS |
|  |  | Received ≤48 h after onset | 49/328 (15) | 10/93 (11) | 7/67 (10) | 18/101 (18) | 10/44 (23) | 4/23 (17) | NS |
|  | Secondary bacterial infection^§§^ | | 69 (13) | 19 (13) | 14 (13) | 19 (11) | 12 (17) | 5 (12) | NS |
|  | Sepsis | | 276 (51) | 77 (52) | 55 (51) | 89 (52) | 36 (50) | 19 (45) | NS |

Abbreviations: NS, not statistically significant; COPD, chronic obstructive pulmonary disease; CP, cerebral palsy; PD, Parkinson's disease; GERD, gastroesophageal reflux disease; ICU, intensive care unit

* Chi-square test

† Conditions listed are not mutually exclusive; some patients have multiple underlying chronic diseases

‡ Includes bronchiectasis, bronchiolitis, bronchiolitis obliterans organizing pneumonia, cystic fibrosis, Eisenmenger's syndrome, history of pulmonary emboli, hypoventilation syndrome, interstitial lung disease, pulmonary fibrosis, pulmonary hypertension, restrictive lung disease, Sjögren's syndrome, and status post lung transplant

§ Includes pituitary, thyroid, and adrenal disorders

¶ Includes immunosuppressive drugs, induction chemotherapy, cancer, and leukemia.

# Includes adrenal disease, congenital immunodeficiency, and status post splenectomy

** Includes Arnold-Chiari malformation, hepatic encephalopathy, microcephaly, myasthenia gravis, multiple sclerosis, and peripheral neuropathy

†† For cases with extreme obesity (BMI ≥40), 454 (all cases with height and weight data available) was used as the denominator. For obesity (BMI 30-39), 356 was used as the denominator; extremely obese cases (n=98)were excluded in order for the trend test to assess any association between age and the presence of obesity (BMI 30-39) as compared to the absence of obesity (BMI<30)].

‡‡ Includes chronic hepatitis/cirrhosis, diverticulosis/diverticulitis, gastritis, gastroparesis, history of gastrointestinal bleed, history of small bowel obstruction, pancreatic insufficiency, status post gastronomy tube placement, and ulcerative colitis

§§ Includes *Staphylococcus aureus* of all susceptibility patterns (32), *Streptococcus pneumoniae* (13), *Aspergillus* (6), *Streptococcus pyogenes* (5), gram-negative rods NOS (4), *Acinetobacter baumanii* (3), *Candida albicans* (2), *Pseudomonas aeruginosa* (2), *Klebsiella* (2), *Candida tropicalis* (1), *Enterococcus* (1), *Stenotrophomonas maltophilia* (1), *Streptococcus agalactiae* (1), *Enterobacter cloacae* (1), and *Streptococcus viridans* (1)
